# Recent Advances in SELEX Technology and Aptamer Applications in Biomedicine

**DOI:** 10.3390/ijms18102142

**Published:** 2017-10-14

**Authors:** Zhenjian Zhuo, Yuanyuan Yu, Maolin Wang, Jie Li, Zongkang Zhang, Jin Liu, Xiaohao Wu, Aiping Lu, Ge Zhang, Baoting Zhang

**Affiliations:** 1 School of Chinese Medicine, The Chinese University of Hong Kong, Hong Kong 999077, China; zhenjianzhuo@163.com (Z.Z.); lijie_bio@126.com (J.L.); maxzhangzk@cuhk.edu.hk (Z.Z.); 2 Law Sau Fai Institute for Advancing Translational Medicine in Bone & Joint Diseases, School of Chinese Medicine, Hong Kong Baptist University, Hong Kong 999077, China; yu.yy01@hotmail.com (Y.Y.); wangml1240@163.com (M.W.); liujin_hkbu@163.com (J.L.); wxho0606@163.com (X.W.); 3 Institute of Integrated Bioinfomedicine and Translational Science, School of Chinese Medicine, Hong Kong Baptist University, Hong Kong 999077, China; 4 Institute of Precision Medicine and Innovative Drug Discovery, School of Chinese Medicine, Hong Kong Baptist University, Hong Kong 999077, China; 5 Shenzhen Lab of Combinatorial Compounds and Targeted Drug Delivery, HKBU Institute of Research and Continuing Education, Shenzhen 518000, China

**Keywords:** aptamer, SELEX, selection, targeted drug delivery systems, therapy

## Abstract

Aptamers are short DNA/RNA oligonucleotides capable of binding to target molecules with high affinity and specificity. The process of selecting an aptamer is called Systematic Evolution of Ligands by Exponential Enrichment (SELEX). Thanks to the inherit merits, aptamers have been used in a wide range of applications, including disease diagnosis, targeted delivery agents and therapeutic uses. To date, great achievements regarding the selection, modifications and application of aptamers have been made. However, few aptamer-based products have already successfully entered into clinical and industrial use. Besides, it is still a challenge to obtain aptamers with high affinity in a more efficient way. Thus, it is important to comprehensively review the current shortage and achievement of aptamer-related technology. In this review, we first present the limitations and notable advances of aptamer selection. Then, we compare the different methods used in the kinetic characterization of aptamers. We also discuss the impetus and developments of the clinical application of aptamers.

## 1. Introduction

Aptamers are a class of agents that act as affinity probes or molecular recognition elements for diagnostic or therapeutic purposes. They are synthetic single-stranded DNAs or RNAs, which bind to target molecules with high affinity in three-dimensional shapes. The term ‘aptamer’ was derived from the combination of the Latin word aptus (‘to fit’) and the Greek word meros (‘part’). The process of aptamers’ selection, termed Systematic Evolution of Ligands by Exponential Enrichment (SELEX), was first developed in 1990 by Tuerk and Gold [1] and Ellington and Szostak [2], separately. To date, thousands of aptamers targeting amino acids, proteins, small metal ion, organic molecules, bacteria, viruses, whole cells and animals have been generated [3]. These aptamers have been widely applied in analytical, bioanalytical, imaging, diagnostic and therapeutic fields. The following factors might contribute to the binding affinity between aptamers and their targets: hydrogen bonding, structure compatibility, stacking of aromatic rings, electrostatic and hydrophobic interactions and van der Waals forces [4].

Aptamers are highly comparable to antibodies, with the characteristics of binding to their targets with high affinity and specificity. Due to the advantages of the aptamers against antibodies listed in Table 1, aptamers have been increasing in popularity since their appearance. It is reported that more than 2000 aptamers have been generated during the past 30 years [5]. Despite great achievements, there still exists a larger lag in the successful clinical use of therapeutic aptamers than that of therapeutic antibodies. Only one aptamer, pegaptanib (Macugen; Pfizer/Eyetech), has been approved for clinical use [6]. Moreover, this Vascular Endothelial Growth Factor (VEGF)-targeted aptamer still fails to surpass anti-VEGF monoclonal antibodies such as ranibizumab (Lucentis; Genentech, San Francisco, CA, USA) and bevacizumab (Avastin; Genentech) in therapeutic effects [7]. Currently, two main barriers are attributed to the impediment of aptamer development and application. First, the SELEX process is still time consuming, and the successful rates are low. Second, most of the current aptamers are obtained in vitro, and whether they can function in vivo needs to be elucidated.

Herein, we first highlight the limitations and technological advances in the selection of aptamers. Then, the applications of aptamer for diagnosis in biomedicine are discussed. We also offer an overview of the clinical application of aptamers. We hope that this review will provide a better understanding for the development of aptamers.

## 2. Limitations and Advancements in Aptamer Selection

**Figure 1:**
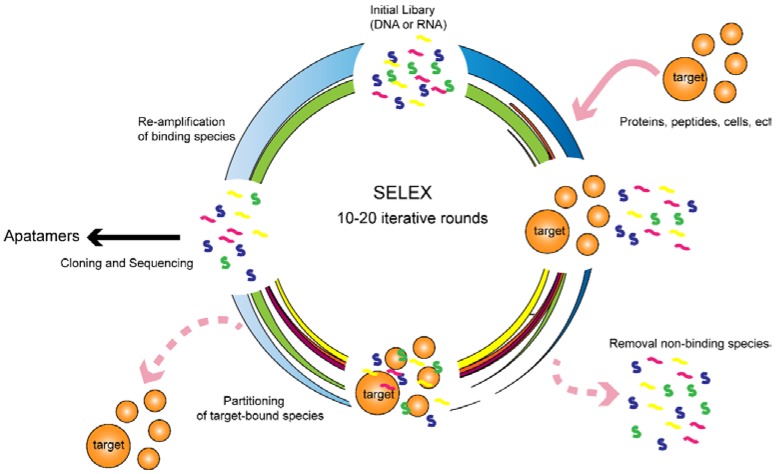
Schematic illustration of the SELEX process for the DNA and RNA library.

In 1990, two laboratories independently developed SELEX. Since then, great modifications and improvements have been achieved. The conventional SELEX method mainly consists of the following three steps: selection, partitioning and amplification (Figure 1). Before the selection, a library of oligonucleotides is synthesized, which generally contains up to 10^15^ different unique sequences [1]. Each unique sequence contains random bases (20–50 nt) flanked by two conserved primer binding sites, which are used for PCR amplification by annealing primers. In the selection step, the library is incubated with target molecules for the indicated time. After incubation, the unbound sequences are separated from those that bound by different methods. The target-bound sequences are amplified by PCR (DNA SELEX) or reverse transcription PCR (RNA SELEX). The PCR products, being a new sub-pool, are utilized for the next round of selection. After several selection rounds, the enriched sequences are sequenced, and their binding abilities are further evaluated. Generally, it takes from weeks to months to obtain specific aptamer candidates, and the hit rates are low. Thus, the obtainment of high quality aptamers against relevant targets still remains a bottleneck. To shorten the selection time and enhance the hit rates, several modified SELEX methods have been set up.

### 2.1. Negative SELEX

Generally, the target molecules are attached to the immobilization matrix, which enables partition. During the selection period, some of the sequences might unwantedly bind to the immobilization matrix, causing false positive results. To eliminate such a possibility, Ellington and Szostak put forward a new method called negative SELEX in 1992 [8]. After three selection cycles, they incubated the library with purification support agarose as negative selection. The non-specific binding sequences were removed from each pool. The affinity of the resultant aptamers was about 10-times higher than that of aptamers obtained without negative selection. After that, most of the modified SELEX incorporated this process to eliminate nonspecific sequences generated by their binding to the immobilization matrix.

### 2.2. Counter SELEX

Counter SELEX is similar to negative SELEX in procedure and purpose, except that counter SELEX uses similar target molecules as incubation subjects. The counter SELEX method was introduced by Jenison et al. in 1994 to enhance the specificity of aptamers [9]. Compared to traditional SELEX, counter SELEX adds an additional step using structurally-similar targets to incubate with aptamers to effectively discriminate non-specific oligonucleotides. This procedure has been dramatically applied to other modified SELEX methods to obtain more specific aptamers [10,11,12]. It is to be noted that the prominent difference between counter SELES and negative SELEX is their use of different incubation objects.

### 2.3. Capillary Electrophoresis SELEX

In general, it takes about more than 15 rounds to obtain aptamers using the conventional SELEX method, which is labor intensive and time consuming. In 2004, a modified SELEX method called Capillary Electrophoresis SELEX (CE-SELEX) was developed [13,14]. CE-SELEX separates the target bounded sequences from unbound sequences by the difference in electrophoretic mobility, which is a highly efficient separation method. This method enables the selection of aptamer candidates with high affinity, while reducing the selection rounds to 1–4 from nearly 20 in conventional SELEX. Up to now, several aptamers have been successfully selected using CE-SELEX [15,16].

In order to further accelerate the selection procedure and to minimize the DNA amplification bias caused by the repetitive steps of PCR, modified selection methods have been developed. Another CE-based technology, which is called non-SELEX, selects an aptamer without amplification [17]. This technology utilizes Non-Equilibrium Capillary Electrophoresis of Equilibrium Mixtures (NECEEM), a highly efficient affinity method, to partition the oligonucleotides-target complex from the free oligonucleotides. Non-SELEX further shortens the selection time to one hour in contrast to several weeks or months required for a conventional SELEX. This method has been successfully adopted to select aptamers against bovine catalase [18]. However, the limited volume of the library injected into the capillary limits the number of sequences (~10^12^ sequences) [13].

To overcome the limited size of the library, Jing et al. [19] modified this method by using micro Free Flow Electrophoresis (μFFE). The starting sequences in a library can reach up to ~10^14^, a 300-fold improvement in library size over CE-SELEX. However, only one aptamer was obtained using this method, which might be due to the need for the fabrication of special μFFE equipment.

### 2.4. Microfluidic SELEX

In 2006, Hybarger et al. developed a microfluidic SELEX (M-SELEX) prototype by combining traditional SELEX with a microfluidic system [20]. The prototype contains reagent-loaded micro-lines, a pressurized reagent reservoir manifold, a PCR thermocycler and actuatable valves for selection and sample routing. Using this device, they successfully obtained an RNA aptamer against lysozyme. In 2009, Luo et al. described a more efficient, rapid and automatic aptamer selection system [21]. This system integrates the magnetic bead-based SELEX process with microfluidics technology and a continuous-flow magnetic activated chip-based separation device. Only after a single round of selection, an enriched aptamer pool was obtained that could bind to recombinant botulinum neurotoxin type A with high affinity. However, the magnetic beads in the microchannel might aggregate together, thus causing low aptamer purity and recovery. Besides, microbubbles in the microchannel might distort the flow streams. To overcome these disadvantages, Soh et al. further improved the M-SELEX by fabricating the microchannel with ferromagnetic materials. They obtained aptamers targeting streptavidin with a Kd value of 25 nM in only three rounds of selection using this improved M-SELEX.

Another novel microfluidic SELEX incorporating nanoporous sol-gel protein microarray material was developed by Park et al. in 2009 [22]. The nanoporous structure of sol-gels can hold a large number of target molecules, which enables the selection of aptamers against multiple target molecules. Several target molecules with Kd in the low nM range have been generated using sol-gel SELEX. The selection efficiency of aptamers is elevated with the reduction of selection cycles to 5–8 rounds [23,24,25]. However, the concerns of protein integrity and stability through the multiple selection cycles in sol-gel SELEX still exist.

More recently, several modified microfluidic techniques have been established to enhance the efficiency in selecting aptamers, including Capillary Electrophoresis (CE) microfluidic SELEX [26], bead-based microfluidic SELEX [27,28] and protein microarray-microfluidic chip SELEX [29].

### 2.5. Cell SELEX

In vitro SELEX has obtained many aptamers targeting purified proteins, but most of these aptamers might not be able to bind to the same proteins at endogenous levels or conditions in cells. Cell SELEX employs the whole live cells as the target, which increases the possibility of the selected aptamer to be used directly for diagnostic and therapeutic applications. Compared to in vitro SELEX, cell SELEX has many merits: first, molecular targets on the surface of the cells are in their native conformation; the aptamers obtained represent the eventual results; second, there is no need for protein purification or prior knowledge of the molecular targets on the cell surface before selection; third, this method could be used to discover new biomarkers or some unknown surface proteins.

In 2003, Daniels et al. firstly developed the cell SELEX method, and they successfully obtained a DNA aptamer against tenascin-C using a glioblastoma-derived cell line, U251 [30]. Currently, several modified cell SELEX methods have been established to improve the success rate of aptamer screening. In 2001, Hicke et al. developed a hybrid SELEX that combines the advantage of cell SELEX and purified protein-based SELEX [31]. They first incubated the library with tenascin-C-bearing cells to select aptamer candidates against tenascin-C in its native conformation. Then, they performed the following rounds of selection against purified tenascin-C protein. In most cases, purified recombinant proteins are used as targets for selection. However, it is time consuming and difficult to purify some cell surface proteins. To overcome this issue, Ohuchi et al. designed a novel SELEX method, termed TECS SELEX, in which a cell-surface displaying recombinant protein was directly used as the selection target [32]. They successfully obtained RNA aptamers towards the transforming growth factor-β type III receptor expressed on Chinese hamster ovary cells. This method has been further approved by Chen et al. to obtain aptamers against dendritic cell-specific intercellular adhesion molecule-3-grabbing nonintegrin (DC-SIGN) displayed on the surface of NIH3T3 cells [33]. Recently, Soldevilla et al. developed another hybrid SELEX combining cell SELEX and peptide SELEX. They successfully obtained an aptamer that targets chemotherapy-resistant tumors expressing MRP1 [34]. Other cell SELEX-based methods such as FACS-SELEX [35,36], 3D cell SELEX [37] and cell-internalization SELEX [38,39] were also developed.

### 2.6. In Vivo SELEX

Considering that aptamers selected in vitro may not always be functional in vivo, researchers developed an in vivo-based SELEX method to generate tissue-penetrating aptamers directly within animal models of the target diseases. In 2010, Mi et al. firstly tried to select aptamers inside a tumor of a living organism [40]. The procedure of this in vivo SELEX is similar to that of traditional SELEX, except for the selection target. In brief, a library of 2’-fluoropyrimidine-modified RNA aptamers was injected into the tail vein of intrahepatic tumor-bearing mice. After that, the aptamers were extracted from the liver tumors, amplified and re-injected into other mice bearing the same tumor. They successfully selected aptamers against p68 and RNA helicase with Kd values in nano-molar levels. In an attempt to identify aptamers that can penetrate through the blood-brain barrier, Cheng et al. injected a 2’-fluoropyrimidine-modified RNA library into the mice [41]. Using this method, they identified aptamers that could bind to brain capillary endothelia and penetrate into the parenchyma. These examples demonstrate that it is feasible to generate aptamers using live-animal model as selection targets or conditions.

### 2.7. High-Throughput Sequencing SELEX

Up to now, the main method to identify the individual sequence of the final enriched library has been using classic Sanger sequencing analysis. In most cases, the final library contains thousands of sequences, for which it is hard to identify which one is the best aptamer. In addition, the most abundant sequences of the final selection round are not always the ones with high affinity and specificity. Recently, High-Throughput Sequencing (HTS) technology was introduced to the SELEX procedure. The most predominant characteristics of HTS-SELEX are that it firstly allows for sequencing the library across all the selection rounds. Thus, enriched sequences are visible at a much earlier round, which is more time efficient. Fewer selection rounds also avoid the potential PCR bias caused by over selection. In addition, global analysis of large sequence datasets by robust bioinformatics tools can further facilitate comprehensive characterization of aptamers, including binding affinity and/or specificity, structure prediction, abundance quantification and aptamer-target interactions [42]. The first application of high-throughput sequencing in SELEX was performed by Cho M et al. in 2010. They identified aptamers that specifically bind to PDGF-BB protein with K_d_ < 3 nM within three rounds [43]. In 2012, Berezhnoy et al. also adopted HTS-SELEX to identify high affinity aptamers against the IL-10 receptor after five rounds of selection [44]. Since then and especially in the last five years, several aptamers against different targets have been identified using HTS-SELEX [45,46,47,48].

## 3. Kinetic Characterization of Aptamers

It is critical to characterize the binding properties of aptamers before applying them to further research. Understanding key binding properties of aptamers, including affinity, kinetics, specificity and buffer sensitivity, supports aptamer design and use. Several biophysical instruments or techniques have been developed to measure aptamer-target binding affinity.

Isothermal Titration Calorimetry (ITC) is a label-free method that adopts thermodynamic-related techniques. ITC has been widely used to determine the affinity, stoichiometry and thermodynamic parameters of molecular interactions by measuring heat released from aptamer-target complex formation [49]. However, this method requires relatively large sample quantities and is easily affected by temperature [50].

Another recently developed thermodynamic based-technique is Microscale Thermophoresis (MST) [51]. It is a technique that employs the physical phenomenon of the thermophoresis-movement of molecules in a temperature gradient. The movement phenomenon of molecules against the temperature gradient will result in the depletion of a biomolecule in the heated spot. Using a titration approach, MST measures the affinity constants of a wide variety of interactions in the binding equilibrium.

Surface plasmon resonance (SPR) technology is a high-throughput, real-time and label-free platform used to characterize both the affinity and kinetic properties of aptamer and its target. Typically, either the target or aptamer is first immobilized onto the surface of a sensor chip, then various concentrations of non-tethered analytes are flowed through. Then, the changes in refractive index resulting from the formation of the binding complex are recorded to determine the binding affinity [52]. However, one of the shortages of SPR is its requirement of immobilization of the ligands on the surface of a sensor chip. Such immobilization will influence the binding ability of each.

Flow cytometry is a laser-based method with the capacity to characterize the binding ability of aptamers and targets, which is widely used to detect the binding ability of aptamers and whole cells [53]. The aptamer library is labeled with a fluorescence dye such as FITC dye and then incubated with the target cell. The intensity of the fluorescence indicates the binding affinity of the aptamer to the targeted cell [54]. One of the most predominant characteristics of flow cytometry is its ability to look at the binding of aptamers to their targets in its native conformational state. Thus, it is beneficial to perform this assay with the view toward developing aptamers into therapeutics.

We have created a table (Table 2) with details to compare the different characteristics of bioanalytical techniques for kinetic characterization of aptamers.

## 4. Applications of Aptamers in Biomedicine

### 4.1. Aptamers for Biomedical Diagnostics

Due to the high affinity to their targets, aptamers can be developed as diagnostic tools. In addition, aptamers possess the advantages of easy conjugation and labeling features, which enable them to be easily combined with other novel techniques, such as endogenous nucleic acid analysis, microfluidic cell separation, flow cytometry or nanoparticle-based sensing to maximize their diagnostic functions. The first aptamer used as a diagnostic tool was developed in 1999 by Bruno et al. They used the aptamer selected against *Bacillus anthracic* spores to detect anthrax spores [64]. To date, aptamers have been widely applied for the diagnosis of ophthalmology, cardiovascular diseases or cancer diseases. As an example, Wan et al. took advantage of aptamers against epidermal growth factor receptor (EGFR), a common oncogene that is over-expressed in many cancer types, to identify cancer cells [65]. They immobilized anti-EGFR RNA aptamers on the surface of modified glass and observed that these surface-immobilized EGFR aptamers could specifically capture glioblastoma cells with high specificity and sensitivity. These results indicated that aptamers could be used for the detection of tumor cells or used for the early prognosis of metastasis of cancer.

In general, the aptamer-based diagnostic tools contain two domains: one is the targeting domain (aptamer), and the other is the signaling domain (radionuclide or fluorescent). Li et al. selected a DNA aptamer (XL-33) that could target metastatic colon cancer cells with a Kd value of 0.7 nM [66]. They further truncated the aptamer and labeled it with fluorescein amidite (FAM) aiming to image the cancer tissue. This conjugation displayed a higher detection rate against colon cancer tissue with regional lymph node metastasis (81.7%) than that of non-metastatic colon cancer tissue (66.7%). To profile the features of recently-developed aptamers as diagnostic tools for biomedical uses, some representative aptamers are listed (Table 3).

### 4.2. Aptamer or Targeted Drug Delivery

An aptamer can also be used as a targeted drug delivery system, mainly attributed to its specific binding to a target molecule or an intended site. Moreover, other desirable properties of aptamers, such as high binding affinity and specificity, low immunogenicity and easily modified chemical structure, also make them suitable for superior targeted drug delivery system candidates. Depending on the delivered agents by aptamers, aptamer-targeted drug delivery systems can be classified as three major categories: aptamer-small molecule conjugated systems, aptamer-RNA conjugated systems and aptamer-nanomaterial conjugated systems.

#### 4.2.1. Aptamer-Small Molecule Conjugated Systems

Traditional chemotherapeutic drugs usually lack selectivity, resulting in severe side effects. Doxorubicin (Dox) and daunorubicin, the representative anthracycline antibiotics, have been utilized in curing various cancers. However, they could cause severe side effects due to the un-selective intercalating into the double-stranded CG sequences of DNA and RNA. By now, many attempts have been made in developing targeted doxorubicin delivery [72,73,74]. As doxorubicin can intercalate into aptamers, Bagalkot et al. developed an aptamer-doxorubicin physical conjugate [75]. They used a 2’-fluoropyrimidine-modified RNA aptamer that targets the prostate-specific membrane antigen (PSMA), which is mainly expressed on the surface of human prostatic adenocarcinoma (LNCaP) cells. They verified that this conjugate could specifically target the PSMA-expressing LNCaP cells with high affinity and specificity. Huang et al. covalently linked doxorubicin to the sgc8c DNA aptamer, which specifically targets T-cell acute lymphoblastic leukemia cells [76]. As a result, sgc8c-Dox conjugates reduced cellular toxicity to the non-target cells. More recently, Deng et al. synthesized a doxorubicin conjugated aptamer complex (TLS11a-GC-Dox) against HepG2 cells [73].

#### 4.2.2. Aptamer-Nanomaterial Conjugated Systems

Nanomaterials possess several advantages due to their inherent unique physicochemical properties, including an ultra-small size, a huge surface effect and an excellent absorption rate. The relatively large surface area allows them to incorporate multiple targeting ligands or secondary therapeutic reagents, which makes them promising drug delivery carriers.

Gold Nanoparticles (AuNPs) have many excellent features, such as high stability, high biocompatibility, low toxicity and easy modification. Luo et al. designed a smart drug carrier, an aptamer/hairpin DNA-AuNPs conjugate, for targeted delivery of drugs [77]. They firstly assembled the protein tyrosine kinase 7 (PTK7) DNA aptamer sgc8c onto the AuNPs surface. Then, they further loaded the anticancer drug doxorubicin into the repeated d(CGATCG) sequence within the hairpin DNA on the AuNPs surface. Such a conjugated complex could enhance the anti-tumor ability and diminish toxicity. Dam et al. devised a nanoconstruct consisting of an AS1411 anti-nucleolin aptamer and a gold nanostar core [78]. Compared to fibrosarcoma tumors, such a nanoconstruct has a five-times accumulation in invasive breast cancer tumors in a tumor-specific manner without obvious acute toxicity.

Liposomes are another promising candidate for drug delivery. The prominent characteristics of liposomes are their dual hydrophobic and hydrophilic characteristics, which make them capable of encapsulating hydrophilic or hydrophobic drugs. Besides, the surface of the liposome can also be attached with various ligands like PEG, prolonging its in vivo circulation time and therefore enhancing its accumulation at the target sites. Indeed, some liposome-based conjugates have been approved for clinical disease treatment by the U.S. FDA [79,80]. To date, a growing number of research works has been conducted to expand the application of liposomes in combination with aptamers.

In 2009, Cao et al. for the first time devised an aptamer-liposome delivery system [81]. They first attached a 12-thymine spacer to the 3’-end of the anti-nucleolin aptamer sequence. After that, the spacer was further attached with a cholesterol tag for the immobilization on a PEGylated liposome hydrophobic surface, and the chemotherapeutic drug cisplatin (to induce anti-proliferation activity) or the hydrophilic dye calcein (to monitor internalization) were encapsulated into the liposome core. They found that this conjugate could deliver cisplatin in a cancer cell-specific manner. Alshaer et al. conjugated a 2’-F-pyrimidine modified RNA aptamer against CD44 receptor protein to the surface of PEGylated liposomes [82]. They found that the conjugate showed significantly enhanced binding to CD44-expressing cancer cells compared to that of blank liposomes. More recently, Stuart et al. constructed a unique aptamer-functionalized liposomal complex, to deliver zinc chelator, *N*,*N*,*N*’,*N*’-Tetrakis(2-Pyridylmethyl)-Ethylenediamine (TPEN), to prostate cancer cells [83]. The authors demonstrated that TPEN could be specifically delivered to the prostate cancer cell in vitro and in vivo. In addition to the nanomaterials mentioned above, block polymeric nanoparticles, carbon nanotubes, gold-magnetic nanoparticles, Quantum Dot (QD) serum albumin nanoparticles and dendrimers are also being used to combine with aptamers as a targeted delivery.

#### 4.2.3. Aptamer-RNA Conjugated Systems

RNA interference (RNAi) therapy is an ideal strategy widely used for the treatment of diseases. However, the lack of selectivity and specificity for this therapy hampers the successful translation of its broad clinical application. Thus, aptamers to be linked with microRNA (miRNA), small interfering RNA (siRNA) and small hairpin RNA (shRNA) have been extensively explored for targeted delivery of these functional RNAi into the target location.

The first aptamer-siRNA chimera was proposed by Mcnamara et al. in 2006 [84]. They covalently linked an RNA aptamer against human PSMA (termed A10) to therapeutic siRNAs targeting the two survival genes PLK1 and BCL2 that are overexpressed in many human cancers. The chimeras specifically target prostate cancer cells expressing PSMA and silence the target gene of siRNA, which led to specific inhibition of tumor growth in a xenograft model of prostate cancer. They further modified the chimera to improve the knockout efficiency and the in vivo stability following intravenous injection in subsequent research [85]. It is also worth noting that our team developed CH6 aptamer-functionalized Lipid Nanoparticles (LNPs) encapsulating osteogenic Pleckstrin homology domain-containing family O member 1 (Plekho1) siRNA (CH6-LNPs-siRNA) [86]. The in vitro results showed that specific osteoblasts’ aptamer CH6 can facilitate the knockdown efficiency of Plekho1 siRNA. Moreover, in vivo administration of CH6-LNP-siRNAs facilitated the specific accumulation of siRNAs in osteoblasts and increased bone anabolism in ovariectomized rats. This progression might contribute to the clinical translation of osteoblast-specific aptamer-RNAi-based treatments for bone disease. Nowadays, targeted siRNA delivery using an aptamer-siRNA chimera is being actively studied [87,88,89].

miRNAs are another represented class of aptamer-oligonucleotide therapeutics. In many cases, deregulation of miRNA expression is accompanied with disease. Thus, the restoration of miRNA levels by specific delivery has attracted attention. Esposito et al. developed an aptamer-miRNA conjugate, termed “GL21.t-let” [90]. The aptamer (GL21.t) could specifically bind to and antagonize the oncogenic receptor tyrosine kinase Axl and the human let-7g miRNA as a gene-silencing moiety. They found that the conjugate could selectively delivery let-7g miRNA to target cells and silence let-7g target genes. In addition, the conjugate reduced tumor growth in a xenograft model of lung adenocarcinoma. Recently, they further designed a combined aptamer-miRNA-based targeted system to eliminate glioblastoma stem-like cells [91]. Two aptamers against Axl and PDGFRβ, respectively, were selected as carriers for miR-137 and antimiR-10b. They demonstrated that these two molecular carrier aptamers could deliver miR-137 and antimiR-10b to the glioma stem cell population, which resulted in the inhibition of glioblastoma stem-like cells’ propagation.

The shRNA was also used to conjugate to aptamers. Soldevilla et al. firstly identified high-affinity aptamers against CD40 and further conjugated CD40 agonist aptamer-shRNA chimera to target the inhibition of the Nonsense mRNA-Mediated Decay (NMD) to tumor cells. They demonstrated that such a chimera could lead to significant longer overall survival of B-cell lymphoma-bearing mice [92].

Aptamer-aptamer conjugated systems are gaining attention now. Brett Schrand et al. found that systemic administration of the VEGF-targeted 4-1BB aptamer conjugates engendered potent antitumor immunity against multiple unrelated tumors and exhibited a superior therapeutic index compared with nontargeted administration of an agonistic 4-1BB Ab or 4-1BB aptamer [93]. Fernando Pastor et al. also showed that bi-specific oligonucleotide aptamer conjugates (4-1BB aptamer ligand-PSMA aptamer conjugates) can deliver costimulatory ligands to tumor cells in situ and enhance antitumor immunity [94]. Their group also developed MRP1-CD28 bi-specific oligonucleotide aptamers to target costimulation to drug-resistant melanoma cancer stem cells [34].

### 4.3. Application of Aptamers in Therapeutics

One of the most important applications of aptamers is being used as small-molecule therapeutic agents. Generally, aptamers used for therapy function in two ways. Either aptamers can function as antagonists by specifically binding to target proteins and inhibiting protein-protein interactions or function as agonists by increasing the ability of the target proteins. The first SELEX experiment was designed by Tuerk and Gold in 1990, in which an RNA aptamer against bacteriophage T4 DNA polymerase was obtained [1]. Since then, numerous aptamers have been obtained. To date, only one aptamer, called Macugen, was approved for clinical use by the FDA in treating neovascular wet Age-related Macular Degeneration (AMD). In addition, ten other aptamers are now being evaluated in clinical trials for the treatment of various conditions, including cancer, macular degeneration, coagulation and inflammation (Table 4). The details of these therapeutic aptamers under clinical trials can be referenced in [3,95].

## 5. Challenges of Aptamer-Based Therapeutics

Although nucleic acid aptamers have many merits, their inherent physicochemical characteristics such as short half-lives, susceptible nuclease degradation and rapid renal filtration excretion have limited the in vivo therapeutic potency of aptamers. To remove the barriers of the application of aptamer-based therapeutics, modifications and conjugations of aptamers have therefore been developed.

### 5.1. Nuclease Degradation

The in vivo half-lives of unmodified aptamers are generally less than 10 minutes due to the nuclease-mediated degradation [96]. Therefore, several strategies have been established to resist nuclease degradation. In general, the modification sites are: (1) ends of nucleic acid chain, (2) sugar ring of nucleoside and (3) phosphodiester linkage. Most aptamers in clinical studies are chemically modified by capping the 3’ end with inverted thymidine, such as Pegaptanib, REG1, ARC1779, ARC1905 and BAX499 [97,98,99]. Similar to the 3’-end with inverted thymidine, the 3’-end with inverted biotin is another strategy to resist degradation of 3’-exonuclease. Dougan et al. found that the linkage of 3’-biotin-streptavidin to the thrombin aptamer could enhance resistance of the digestion of 3’-exonuclease in the blood of rabbits or mice [100]. Modifications on the sugar ring generally involve: replacing the 2’ position with a fluoro (F), amino (NH2) or *O*-methyl (OCH3) groups. A ribonucleotide analog, with the sugar ring linked by a methylene between 2’-O and 4’-C, called Locked Nucleic Acid (LNA), was also incorporated into aptamers [101]. Such a modification greatly enhanced the resistance to nucleases. To enhance the flexibility of the aptamer, another ribonucleotide analog without the C2’-C3’ bond, called Unlocked Nucleic Acid (UNA), was attached to the aptamer [102]. As for the phosphodiester linkage, replacing it with the phosphorothioate or methylphosphonate analog is a common option [103]. A new strategy by converting natural d-form nucleic acids into L-enantiomeric nucleic acids was adopted to synthesize aptamers called Spiegelmers [104]. Such Spiegelmers could prevent nucleases degradation. Up to now, three L-aptamers, NOX-A12, NOX-H94 and NOX-E3, have been evaluated in clinical trials.

As the function of an aptamer depends on its structure, most of the aptamers might suffer from altering properties due to the post-SELEX modification, thereby compromising binding affinity [105]. Therefore, it is necessary to predict the final function of modified aptamers. Unfortunately, universal rules are not available for predicting the optimal modification, and thus, laborious optimization is often needed [106]. Several new polymerases are also used to incorporate modified analogs into aptamers. Padilla et al. used a T7 RNA polymerase double-mutant Y639F/H784A to incorporate modified nucleotides into aptamers [107]. The Sawai group has adopted KOD Dash DNA polymerase to incorporate several chemically-modified nucleotides during PCR [108].

### 5.2. Renal Filtration

The relative small mass (6–30 kDa) and short diameter (<5 nm) of aptamers inevitably makes them easily excreted through renal filtration, even when using stabilizing backbone modifications. Thus, formulation with a bulky moiety helps to overcome renal filtration and extend circulation time. The main strategy of enlarging the aptamer size is to attach a bulky moiety, such as cholesterol [109], polyethylene glycol (PEG) [110], proteins [111], liposomes [112], organic or inorganic nanomaterials [113], at the 5’-end of aptamers. Lee et al. attached cholesterol at a 29 nucleotide-long 2’-F pyrimidine-modified RNA aptamer to form a cholesterol-conjugated aptamer (chol-aptamer). They found that modified aptamers were shown to be highly resistant to nucleases degradation in serum with a longer half-life with toxicity [109]. PEG has been approved by the U.S. FDA in drug use. It is widely used to prolong circulation half-life and improve the in vivo bioavailability of therapeutic drugs. PEG was firstly adopted by Jaschke et al. to extend aptamer half-life in vivo [114]. As the half-life is short for the anti-PD-1 aptamer (<1 h) as a result of the rapid renal filtration, Prodeus et al. further conjugated a 40-kDa PEG at its 5’-end. The found that the PEGylated anti-PD-1 aptamer retained the targeting ability to PD-1, thus blocking the binding of PD-1 to PD-L1 [115]. Yet, there still exist some serious allergic reactions to the PEG group, because of the presence of pre-existing antibodies against PEG [116]. It is reported that some acute illness cases occurred in the phase III study of Regado Biosciences’ aptamer-based anticoagulation system, the REG1 system [117]. Another strategy to increase the molecular mass of the aptamer is to multimerize the single aptamers together. By coupling >2 aptamers into a multi-aptamer complexes, multivalent aptamers have shown an increased retention in circulation [118].

## 6. Conclusions

Since the first introduction of the SELEX method in 1990, remarkable achievements have been made. Up to weeks and months are needed using the traditional SELEX in selecting aptamers. In addition, the success rate for obtaining aptamers with high affinity and specificity is very low. With the introduction of new technology, the selection time is greatly reduced, and aptamers with better affinity and selectivity are more easily obtained.

The entire process of the SELEX using the traditional sequencing method is considered to operate within a ‘black box’, as no information can be known during the selection period. It is worthwhile to mention that high-throughput sequencing-SELEX represents the overall revolution of aptamer selection. High throughput sequencing to selected pools of molecules provides an excellent tool for identifying the best aptamers against particular targets in a much faster and safer way. Moreover, it makes the selection process visible in each round of selection, as well. Despite the fact that high throughput sequencing is far more excellent than cloning and Sanger sequencing, it is not widely used now due to the high cost [119]. In the near future, it will be getting easier and quicker to obtain aptamers with high affinity and specificity for clinical use, with the development of new instruments and software, combining high-throughput sequencing with high-throughput binding analysis. Aptamers are also ideal targeting ligands for targeted therapy, due to their possession of the high affinity and specificity ability. Various aptamer-based drug delivery systems such as aptamer-chemotherapy agents, aptamer-siRNA/shRNA/miRNA, aptamer-antibody, aptamer-enzyme and aptamer-nanoparticles have been established to specifically deliver the drug to the expected sites, therefore reducing the possibility of side effects caused by the off-target effects. Yet, despite great advances having been achieved in aptamer selection and application, few aptamers have come into commercial application successfully. The following reasons might be associated with this embarrassment: (1) most of the aptamers were selected in vitro, and they might not bind to the target in vivo; (2) aptamers are 20–70 nucleotides long with a small molecule size, which result in the ease of their kidney filtration and the short circulation time in vivo; (3) to some extent, the relative mature antibody-based market will impede the development of the relatively new aptamer products, especially the lack of a fundamental revolution in aptamers’ selection. Nonetheless, the attractive advantages of aptamers over antibodies still bring broad prospects for aptamers’ development. With many advances in place, several aptamers are now in clinical trials or in the pipeline. Where the field progresses from here is uncharted; however, it seems clear that the next generation of therapeutic aptamers will be obtained more quickly, require less post-selection stabilization and encompass more functionality. A more detailed review of both the therapeutic hurdles and clinical trials of aptamers was conducted by Sundaram et al. [120].

In summary, with the advances of SELEX technology, new aptamers are being developed in a more efficient way with less cost. It can be expected that more aptamers can be applied to diagnostic and therapeutic purposes in the near future.

## Figures and Tables

**Table 1:** Merits of nucleic acid aptamers over protein antibodies.

Features	Antibody	Aptamer
Materials	Polymer peptide	Nucleic acid
Specificity	High	High
Affinity	High	High
Immunogenicity	High	No humoral response
Production	In vivo	In vitro
Cost	High	Low
Stability	Unstable	Stable
Potential targets	Limited to immunogenic molecules	Wide range
Generation time	~6 months	~3–7 weeks
Modification	Restricted	Convenient

**Table 2:** Summary of bioanalytical techniques for kinetic characterization of aptamers.

Techniques	Description	Advantage	Disadvantage	Refs.
Double-filter binding assay	By using radio labeled DNA, the amount of DNA complexed with protein in a solution can be quantitated by filtering it through nitrocellulose and measuring the amount of radioactivity retained on the membrane	Powerful	Low accuracy	[55,56]
Surface Plasmon Resonance (SPR)	Binding affinity is estimated through changes in the refractive index when binding occurs	High-throughput, label-free, real-time, kinetic information	Require ligand immobilization	[57]
Quartz Crystal Microbalance measurements (QCM)	The frequency of the quartz decreases when immobilized aptamers bind to targets	Label-free, real-time, sensitive, stable	High cost	[58]
UV-Vis	Binding affinity is determined by changes in the maximum absorption with a fixed aptamer concentration, but varying target concentrations	Affordability, label-free	Low accuracy	[59]
Isothermal Titration Calorimetry (ITC)	Heat is measured as the signal, which is released during the formation of the aptamer-protein complex	Versatile	Low throughput, large sample quantities, poor detection limits	[60]
Microscale Thermophoresis (MST)	Binding affinity is measured by the change in mobility caused by the formation of aptamer-target complexes	Rapid, precise	Requires fluorescent labeling	[61]
Flow cytometry	The binding interaction between fluorescence dye-labeled aptamer and target cells is determined from the fluorescence intensity of the labeled cells	Rapid, whole live cell	Requires fluorescent labeling	[54]
Atomic Force Microscope (AFM)	The peak of histogram in the measurement of adhesion at a certain data point is used to detect the binding affinity between aptamer and target	Fixation-free, dehydration-free	High cost	[62]
Backscattering Interferometry (BSI)	The refractive index will change if a binding event occurs and leads to producing a change in the spatial position of the fringes; this fringe shift is monitored using a CCD array in combination with Fourier analysis	Label-free, solution-free, sensitive	High cost	[63]

**Table 3:** Examples of recently developed aptamers for the diagnosis of human diseases.

Name	Target	K_d_ (nM)	Sensitivity	Specificity	Refs.
Cancers					
XL-33	Metastatic colon cancer cells (SW620)	0.7	81.7% (*n* = 71 metastatic colon cancer tissues)	66.7% (*n* = 18 non-metastatic colon cancer tissues)	[66]
yl19	Cholangiocarcinoma cells (QBC-939)	42.4	-	100% (*n* = 6 cancer cell lines)	[65]
LXL-1	Metastatic breast cancer cells (MDA-MB-231)	44.0	76% (*n* = 34)	100% (*n* = 8 cancer cell lines)	[67]
SYL3-C	Solid cancer Epithelial Cell Adhesion Molecule (EpCAM)	22.8	60%	100% (*n* = 3)	[68]
GMT3	Glioblastoma multiforme cells (A172)	75.3	-	87.5% (*n* = 8 cancer cell lines)	[69]
Cardiovascular Diseases					
Myo040-7-27	Myoglobin	4.93	10 pm	-	[27]
Infectious Diseases					
LmWC-25R and LmHSP-7b/11R	Leishmania promastigote and hydrophilic surface protein	-	100 ng (parasite protein)	-	[70]
2008s	*Plasmodium falciparum* lactate dehydrogenase	42–59	57 ng/mL	No human LDH recognition	[71]

**Table 4:** Progress of aptamers for diseases’ therapy in on-going or completed clinical trials.

Name	Form	Target	Condition	Phase
Pegaptanib sodium (Macugen)	27-nt RNA	VEGF (Vascular Endothelial Growth Factor)	Age-related macular degeneration	Approved
E10030	29-nt DNA	PDGF (Platelet-Derived Growth Factor)	Age-related macular degeneration	Phase III
REG1 (RB006 and RB007)	37-nt RNA	Coagulation factor IXa	Coronary artery disease	Phase III
ARC1905	38-nt RNA	C5 (Complement component 5)	Age-related macular degeneration	Phase III
AS1411	26-nt DNA	Nucleolin	Acute myeloid leukemia	Phase II
ARC1779	39-nt DNA	A1 domain of von Willebrand factor	Von Willebrand disease/thrombotic thrombocytopenic/purpura	Phase II
NOX-E36	40-nt RNA	CCL2 (Chemokine C-C motif Ligand 2)	Chronic inflammatory diseases/type 2 diabetes mellitus/systemic lupus erythematous	Phase II
NOX-A12	45-nt RNA	CXCL12 (Chemokine C-X-C motif Ligand 12)	Multiple myeloma and non-Hodgkin lymphoma/autologous or hematopoietic stem cell transplantation	Phase II
NU172	26-nt DNA	Thrombin	Heart disease	Phase II
NOX-H94	44-nt RNA	Hepcidin peptide hormone	Anemia/end-stage renal disease/inflammation	Phase II
ARC19499	32-nt RNA	TFPI (Tissue Factor Pathway Inhibitor)	Hemophilia	Phase I
